# Methodological insights into the dip-and-pull X-ray photoelectron spectroscopy technique: analysing electrochemical interfaces under *in situ/operando* conditions

**DOI:** 10.1107/S1600577525008811

**Published:** 2026-01-01

**Authors:** Benjamin Rotonnelli, Amandine Brige, Alexandr G. Oshchepkov, Jean-Jacques Gallet, Fabrice Bournel, Antoine Bonnefont, Alexander Yaroslavtsev, Andrey Shavorskiy, Robert Temperton, Elena R. Savinova, Tristan Asset

**Affiliations:** a ICPEES, UMR 7515 CNRS-ECPM-Université de Strasbourg, 25 Rue Becquerel, 67087Strasbourg Cedex 2, France; b Sorbonne Université CNRS, Laboratoire de Chimie Physique Matière et Rayonnement (LCPMR) 4 place Jussieu 75005Paris France; c Université Grenoble Alpes, Université Savoie Mont Blanc, CNRS, Grenoble INP (Institute of Engineering and Management Université Grenoble Alpes), LEPMI, 38000Grenoble, France; d MAX IV Laboratory Lund University PO Box 118 SE-221 00Lund Sweden; Bhabha Atomic Research Centre, India

**Keywords:** dip-and-pull XPS, X-ray photoelectron spectroscopy, electrode–electrolyte interface, electrochemistry, *in situ*, *operando*, synchrotron, methodology

## Abstract

Gaining insight into structural and compositional transformations occurring at the electrode/electrolyte interface during the operation of electrochemical systems is fundamental to understanding and, thus, optimizing their performance. Such an analysis must be performed in *operando* conditions, owing to the potential, electrolyte and time dependence of these transformations. Here, the use of X-ray photoelectron spectroscopy (XPS) is particularly attractive due to its surface sensitivity and ability to provide quantitative information on the oxidation state and chemical environment of an element. In specific instrumental configurations [*e.g.* in ‘dip-and-pull’ (D&P) or ‘meniscus’ setup], it can be used to analyse not only the electrode but also the electrolyte side of the interface, under *in situ*/*operando* conditions. In this article, we discuss how D&P XPS can provide unique information on both sides of the electrode/electrolyte interface, briefly review publications demonstrating its capabilities, highlight the challenges the method faces, and share our views on its future developments. This article aims to provide a practical guide to new D&P synchrotron users and help them to understand the technique, and physical phenomena that may impede the acquisition of reliable data.

## Abstract 2

This work provides guidelines on how to meaningfully perform a dip-and-pull X-ray photoelectron spectroscopy (XPS) experiment applied to electrochemistry and electrocatalysis to obtain information on both the electrode properties and the electrode/electrolyte interface. The aim is to facilitate access to the dip-and-pull XPS method to newcomers from the field of electrochemistry.

## 1. Introduction

**Figure 1:**
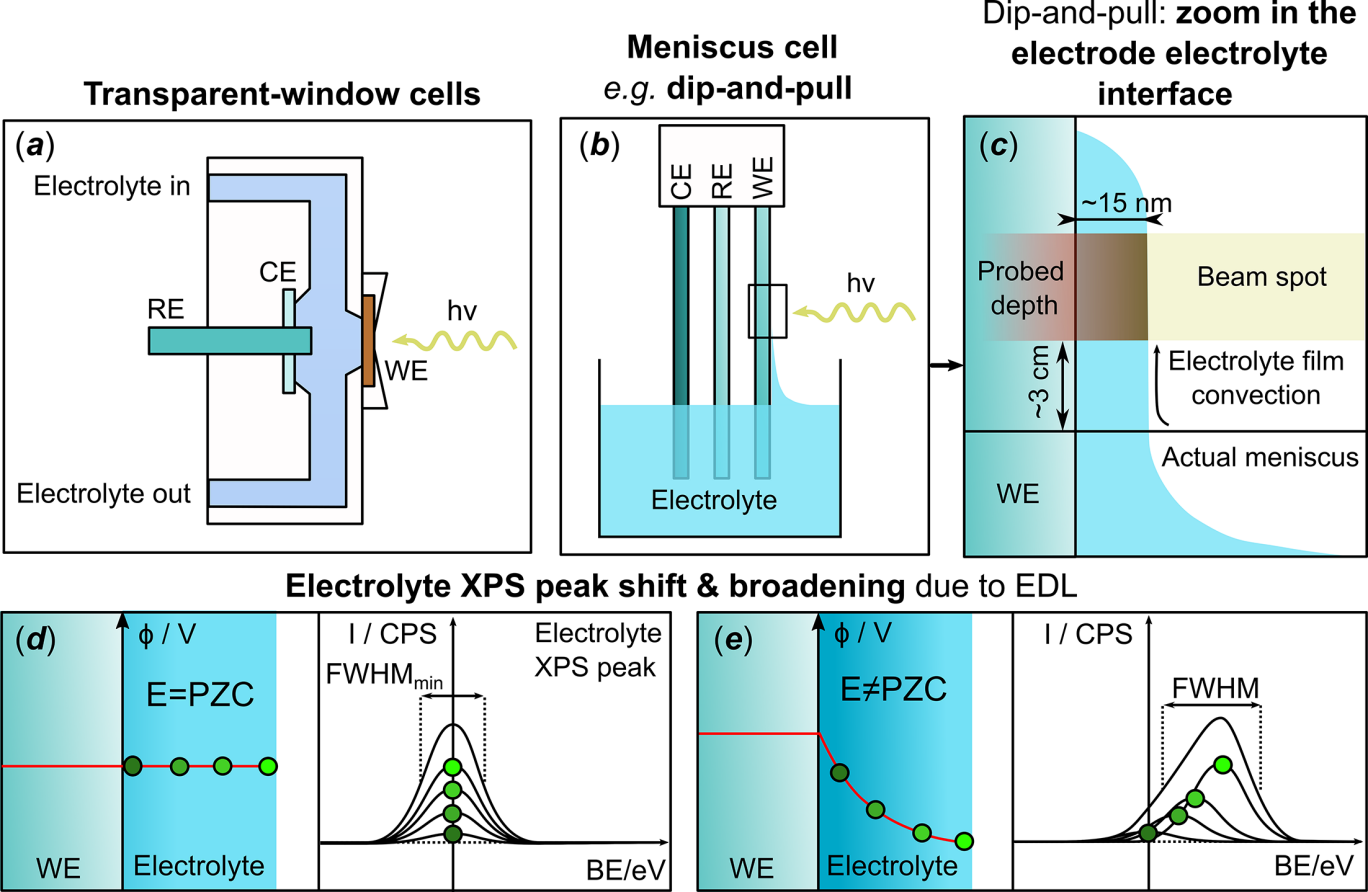
(*a*) Example of XPS *in situ*/*operando* setup for the ‘transparent-window cell’ design; (*b*) D&P spectroelectrochemical cell representation, belonging to the ‘meniscus cell’ type; (*c*) zoomed-in view of the analysed interface; (*d*) and (*e*) correspond to illustration of the XPS signal of dissolved species (*e.g.* cations) in the electrolyte at point of zero charge (PZC) (*d*) and at potential different from the PZC (*e*).

Since the electron transfer between an electrode and a reactant occurs at the electrode/electrolyte interface, electrochemistry is, by definition, an interfacial science. This interface defines reaction pathways, electrocatalytic activity and even durability in electrocatalysis, and performance in energy storage systems (batteries, capacitors *etc*.) (Monteiro *et al.*, 2021 ▸). Therefore, understanding this interface is pivotal. While it is easier to perform characterizations of an electrocatalyst after the reaction, as the setup can simply be dismantled and the sample analysed by a vast array of spectroscopic, microscopic, diffraction and other analytical methods (*e.g.* transmission electron microscopy, scattering electron microscopy, X-ray photoelectron spectroscopy, Raman spectroscopy, X-ray diffraction *etc*.), such a *post-mortem* analysis does not provide information on the electrode/electrolyte interface, as the latter can only be observed under potential control and in the presence of the reaction environment, *i.e. in situ* or *operando* (Maibach *et al.*, 2023 ▸; Atkins *et al.*, 2022 ▸; Handoko *et al.*, 2018 ▸). This is especially evident when it comes to the electrolyte, and/or to the species whose existence is potential, time or environment controlled, and can therefore be altered/destroyed unless they are characterized during the operation of the system. The use of the terms *in situ* and *operando* has been the subject of constant debate over the past decades. To resolve discrepancies in the terminology, IUPAC recently published a technical report (Peterson *et al.*, 2024 ▸) that recommended the utilization of the term *in situ* for a ‘sample at steady state held in a particular environmental parameter within a controlled environment such as a sample environment device’ whereas *operando* stands for ‘sample not at a steady state, under non-equilibrium and non-ambient conditions’. X-ray photoelectron spectroscopy (XPS) is perfectly suited for either *in situ* or *operando* studies probing the interface, as it is element, oxidation state and surface sensitive (Arrigo, 2022 ▸; Rotonnelli *et al.*, 2023 ▸; Källquist, Le Ruyet *et al.*, 2022 ▸). However, the limited inelastic mean free path (IMFP, λ_IMFP_) of photoelectrons in liquid and gaseous media makes collection of X-ray photoelectron (XP) spectra in a (near-)ambient environment challenging. Near-ambient-pressure XPS was pioneered by the Siegbahn group in the early 1970s (Siegbahn & Siegbahn, 1973 ▸) by employing a differential pumping scheme, which has become widespread in the 2000s thanks to technical advances in the electron analysers and the use of intense synchrotron sources [see Starr *et al.* (2013 ▸) and references therein]. To the best of our knowledge, application of the so-called ambient (or near-ambient)-pressure XPS (APXPS or NAP-XPS) to electrochemical systems started out from the study of high-temperature solid-oxide systems, with both the anode and the cathode exposed to a gas atmosphere (Zhang *et al.*, 2010 ▸). A similar approach was then applied to a membrane electrode assembly (MEA) for low-to-intermediate-temperature fuel/electrolysis cells with the whole MEA exposed to the ambient gas (Saveleva *et al.*, 2018 ▸; Pfeifer *et al.*, 2017 ▸; Law *et al.*, 2015 ▸; Arrigo *et al.*, 2013 ▸; Sanchez Casalongue *et al.*, 2014 ▸). As to the cells with a liquid electrolyte, most of the currently applied spectroelectrochemical cells for *in situ*/*operando* XPS measurements can be categorized into two groups, *i.e.* systems relying on (i) transparent-window cells [Fig. 1 ▸(*a*)] or on (ii) meniscus cells [Figs. 1 ▸(*b*)–1 ▸(*c*)].

When addressing the systems within the first category, the main challenge is the structural integrity of the window separating the electrocatalyst from the vacuum, as its degradation would lead to electrolyte leakage in the XPS analysis chamber. Currently, a graphene bilayer, which acts both as a separator and as an electron collector for the working electrode (WE), is often used for that purpose, despite its low mechanical integrity and possible carbon electro-oxidation at high potentials, leading to further mechanical fragility and detachment of the electroactive material (Trotochaud *et al.*, 2017 ▸; Angel *et al.*, 2020 ▸). Mitigating these structural weaknesses can be achieved, *e.g.* by reinforcing a graphene window with a holey SiN_*x*_ (Velasco-Vélez, Falling *et al.*, 2021 ▸; Louisia *et al.*, 2024 ▸; Saveleva & Savinova, 2019 ▸) or deposing the catalyst layer directly on a diaphragm or an ion exchange membrane and covering it with the graphene bilayer to ensure electrical connection, the whole assembly separating the liquid water or electrolyte from the analysis chamber (Hrbek *et al.*, 2024 ▸; Zhang *et al.*, 2024 ▸; Velasco-Vélez, Carbonio *et al.*, 2021 ▸; Royer *et al.*, 2023 ▸; Saveleva, Wang *et al.*, 2016 ▸; Arrigo *et al.*, 2013 ▸; Saveleva *et al.*, 2018 ▸; Pfeifer *et al.*, 2017 ▸).

Nowadays, specialized synchrotron-based end-stations working in the tender X-ray range can support pressures up to 790 mbar (CO + H_2_), or even 2.5 bar in H_2_ atmosphere (Amann *et al.*, 2019 ▸). This recently allowed the development of the remarkable ‘dip-and-pull’ spectro-electrochemical setup (D&P, also named ‘meniscus’ method), which supports the presence of liquid electrolyte in the analysis chamber. The setup is represented in Fig. 1 ▸(*b*). It is similar to a conventional three-electrode cell, where working, counter and reference electrodes are vertically immersed in a liquid electrolyte reservoir, allowing the analysis of a model quasi-2D electrode/electrolyte interface. The method was developed at the Advanced Light Source synchrotron and validated through the study of the surface oxidation of a Pt foil under the *operando* conditions of the oxygen evolution reaction (OER) (Axnanda *et al.*, 2015 ▸). In contrast to transparent-window spectro-electrochemical cells, the D&P method relies on the XPS analysis of the electrode/electrolyte interface through a nanometre-thick electrolyte layer that covers the emersed part of the WE, ensuring availability of ionic species at the analysed spot. Owing to this peculiar geometry, it can also offer information regarding the chemical composition of the electrolyte and the local electrostatic potential as specified below,
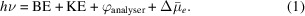
This equation expresses the energy conservation principle for a photoelectron, where *h*ν is the photon energy, BE is the binding energy of the emitted photoelectron, KE is the kinetic energy of the emitted photoelectron after the photon absorption, and φ_analyser_ is the analyser work function. The latter represents the energy difference between the vacuum level of the analyser and the Fermi level, common to the electron analyser and the electrode. Work functions are generally integrated into the acquisition software but an energy reference is needed to obtain an absolute KE/BE energy scale (Fermi level or known core levels are frequently used). In the case of electrochemical interface studies, the photoelectron source (*e.g.* a cation in the electrochemical double layer) may not share the same Fermi level *E*_F_ as the WE. An additional term 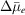, representing the electrochemical potential difference between the photoelectron source and the mass (*i.e*. WE), must be added to account for the difference in energy between the two Fermi levels (Källquist *et al.*, 2021 ▸),
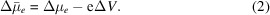
As the electrochemical potential is the sum of the chemical potential and electrostatic potential multiplied by the charge, 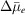 is therefore a sum of the difference between the chemical potential (Δμ_*e*_) and electrostatic potential multiplied by the electron charge (eΔ*V*) [see equation (2)] between the WE and the photoelectron source. Under equilibrium conditions (and in the absence of a Faradaic reaction), 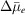 remains null. However, when a local electrical potential is applied, which varies as a function of the distance to the interface upon polarization, as illustrated in Fig. 1 ▸(*e*), the observed KE/BE for the photoelectron sources present in the electrolyte will vary (Boettcher *et al.*, 2021 ▸). In the following we will denote as BE_app_ the apparent BE at which the electrolyte XPS peak appears to be dependent on the applied potential corresponding to the sum of the BE and 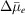. Based on this knowledge, it is important to underline that the shape and the width of the peak could provide insights into the potential distribution at the electrode/electrolyte interface, *i.e.* in the electrochemical double layer [EDL – see Figs. 1 ▸(*d*)–1 ▸(*e*)], which could, if deconvoluted meaningfully, provide information on the potential of zero charge (PZC) of the electrode and the cation distribution in the EDL (Favaro *et al.*, 2016 ▸; Lichterman *et al.*, 2017 ▸).

However, to enable such analysis, D&P strongly relies on the formation of an adequate thin electrolyte film on the surface of the WE. This thin electrolyte film is formed when the WE is first dipped in and then pulled out of the electrolyte, forming a meniscus [Fig. 1 ▸(*c*)]. The electrode is then raised until the film reaches the analysis spot. For the XPS measurement to be sensitive to the electrode/electrolyte interface, the film must remain ultra-thin and below the depth sensitivity (∼3λ_IMFP_) over the whole beam spot. For instance, at the HIPPIE beamline of MAX IV, the analysed spot is ∼60 µm × 25 µm with the depth sensitivity depending on the KE of the photoelectron (∼20 nm for KE = 1.7 keV). Hence, for a successful D&P experiment, an in-depth understanding of the film morphology, integrity, mobility and stability is pivotal. We will elaborate on these aspects in the upcoming sections of the article, using as an experimental basis for the discussion two systems that were investigated by our team at the HIPPIE beamline at MAX IV, namely: (i) a polycrystalline Pt foil in a 1 *M* aqueous CsOH electrolyte with a focus on the electrolyte chemistry (Cs^+^ being preferred to more conventional alkali metal cations such as K^+^ or Na^+^ owing to its higher cross section), (ii) a polycrystalline Ni foil in a 0.1 *M* KOH electrolyte with a focus on the electrode chemistry (since the complex surface chemistry of nickel in an alkaline environment cannot be investigated *ex situ* due to the passivation of Ni under ambient conditions). These experimental illustrations will ground our discussion on the technically and electrochemically induced phenomena occurring while performing a D&P experiment. We hope that the interested reader will find in this article a practical guide to better understand the D&P technique and the pitfalls to avoid, in order to obtain meaningful data.

## 2. Experimental section

### 2.1. Pt/CsOH 1 *M* experiments

**Figure 2:**
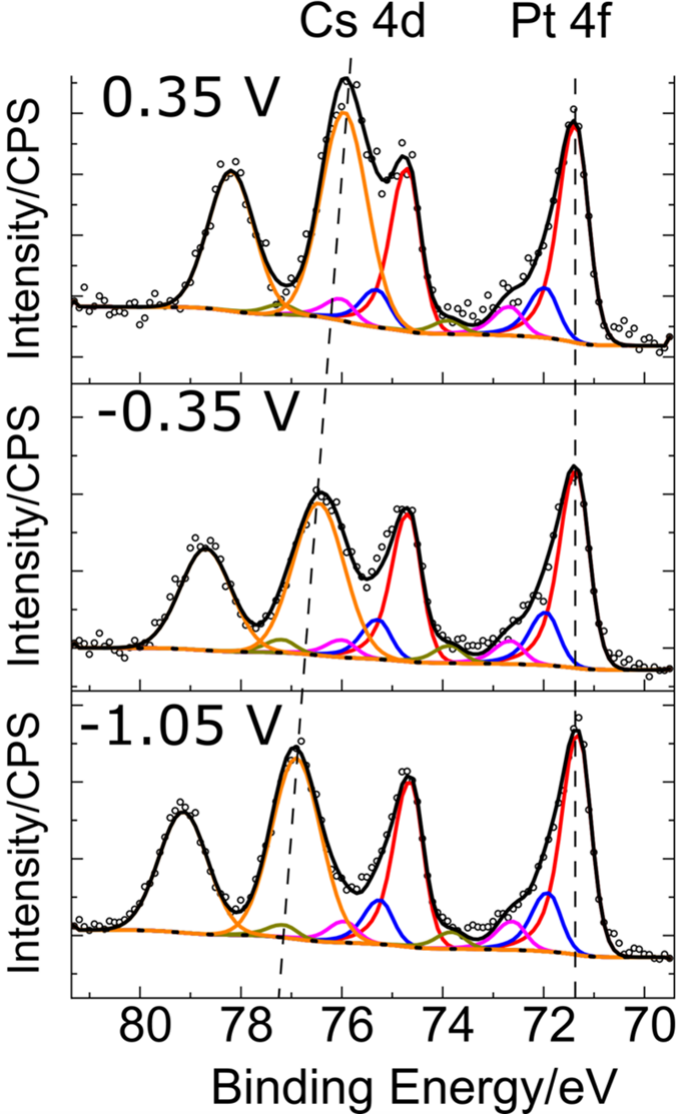
Pt 4*f* and Cs 4*d* XP spectra of the Pt|CsOH 1 *M* electrode|electrolyte interface at different applied potentials. WE = Pt, CE = Pt, RE (all potentials are provided versus RE) = Ag/AgCl. *h*ν = 1800 eV, *P* = ∼15 mbar.

The measurements presented in Figs. 2 ▸–5 ▸ ▸ ▸ were performed at the MAX IV Laboratory on the HIPPIE beamline (Zhu *et al.*, 2021 ▸). For the Pt/CsOH interface study, 1 *M* CsOH electrolyte was prepared by diluting 17.43 ml of CsOH 50 wt% in 100 ml of distilled water. Then, it was degassed in low vacuum using an external vacuum chamber prior to the experiment. Pt foils were used as a WE and counter electrode (CE) while an Ag/AgCl eDAQ leakless mini-electrode was used as the reference electrode (RE). The surface of the Pt foil was (i) polished using diamond paste by successive use of 9 µm, 3 µm, 1 µm and 0.25 µm diamond suspension, then rinsed with water, and (ii) heated to 950°C (10°C min^−1^) to clean the surface. Once assembled on the electrode holder, the RE tip was immersed in the electrolyte to avoid eventual drying of the RE, while the WE was still out of the electrolyte. The analysis chamber was then slowly put under vacuum with pressure controlled down to ∼15 mbar. The pumping station of the analysis chamber was working permanently at a low flow rate to maintain the low pressure and increase the signal intensity. Platinum was first analysed out of the electrolyte, to verify the cleanliness of the surface and to calibrate the analyser using the Pt(0) peak, positioned at 71.3 eV (Saveleva, Papaefthimiou *et al.*, 2016 ▸).

### 2.2. Ni/KOH 0.1 *M* experiments

For the Ni/KOH interface study, a 0.1 *M* KOH solution was prepared from ultra-pure water and KOH pellets (semiconductor grade, 99.99%, Sigma–Aldrich) and bubbled with N_2_ for 30 min to remove traces of dissolved oxygen. Prior to the experiments, a glovebox was mounted in front of the analysis chamber and filled with N_2_. The solution was transferred into the glovebox and then slowly degassed directly in the analysis chamber, to avoid any contamination with O_2_. The WE was a Ni plate with the side of interest polished with alumina powders of decreasing diameter down to 0.3 µm, and the other faces covered by Apiezon Wax W to avoid their contribution to the electrochemical signal. An Ag/AgCl eDAQ leakless mini-electrode was used as a reference, and a roughly polished Ni plate was used as a CE. Before starting the measurements, the Ni WE was polished with 0.3 µm alumina powder to remove the oxide layer, quickly rinsed and immersed in a vial filled with N_2_-saturated ultra-pure water. It was then sonicated for 30 s and quickly introduced into the glovebox. The WE was mounted on the holder together with a small piece of gold foil for calibration purposes. Once introduced in the analysis chamber, the bottom of the electrodes were immersed in a glass beaker filled with KOH electrolyte before decreasing the pressure down to ∼20 mbar.

### 2.3. Data acquisition, treatment and verification procedure

To perform the measurements, the electrode was first dipped in the electrolyte, the desired potential was applied, then the electrode was pulled out until the electrolyte of desired thickness was observed (Fig. S1 in the supporting information). The electrolyte thin-film homogeneity was then verified following the procedure discussed in Section 3 and illustrated in Fig. S2. XPS data were acquired by several short scans to ensure the XPS signal stability over time and the stable signals were summed and fitted (see Fig. S3). Finally, the consistency of the electrolyte thickness and electrode polarization were verified using the ratio between the electrode and the electrolyte XPS signals, and the electrolyte BE XPS signal shift with the applied potential, correspondingly (see Fig. S4).

## 3. Towards an optimization of the D&P XPS operating conditions

To obtain a robust set of results, the electrode/electrolyte interface within the analysis spot must be (i) uniform during the entire experiment, and (ii) present interfacial properties (potential distribution, electrolyte concentration) as similar to a fully immersed electrode/electrolyte interface as possible. It is however not possible to perfectly reproduce the operating conditions of a fully immersed electrode in a D&P system, which is discussed in detail in the following sections. As illustrated in Fig. 1 ▸(*c*), the electrolyte forms a film which extends from the true meniscus at the surface of the electrolyte reservoir with a decreasing thickness, reaching several nanometres on the top. In order to probe both the electrode and the electrolyte species, the analysis spot must be limited to the upper part of the film where the liquid layer is the thinnest, which can extend as far as a few centimetres above the bulk electrolyte surface,
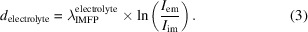
In addition, the electrolyte layer at the analysis spot must be homogeneous and cover the electrode with an adequate and uniform thickness. The latter (*d*_electrolyte_) can be experimentally determined using equation (3) from the electrode XPS peak intensity ratio between the emersed (*I*_em_, without electrolyte – above the electrolyte film) and immersed electrode (*I*_im_, *i.e.* observed through the electrolyte thin film), and the electrons’ IMFP in the electrolyte at the KE associated with the measured peak 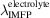 (Axnanda *et al.*, 2015 ▸).

Thus, using equation (3), one can verify if the electrolyte film thickness is constant throughout the experiment. An alternative quick and quantitative approach could be based on the comparison between the intensity of XP peaks originating from the electrolyte on the one hand and the electrode on the other hand. Such an approach is particularly useful and convenient if these peaks concern electrons with close BE values. For instance, on Fig. 2 ▸, representing the D&P XPS signal of a Pt/CsOH 1 *M* interface, we ensured a reproducible ratio between the electrode (Pt 4*f*) and electrolyte (Cs 3*d*) peak intensities in the whole interval of applied potentials. In this way, a reproducible average electrolyte thickness, estimated with equation (3) as ∼13 nm, is obtained.

**Figure 3:**
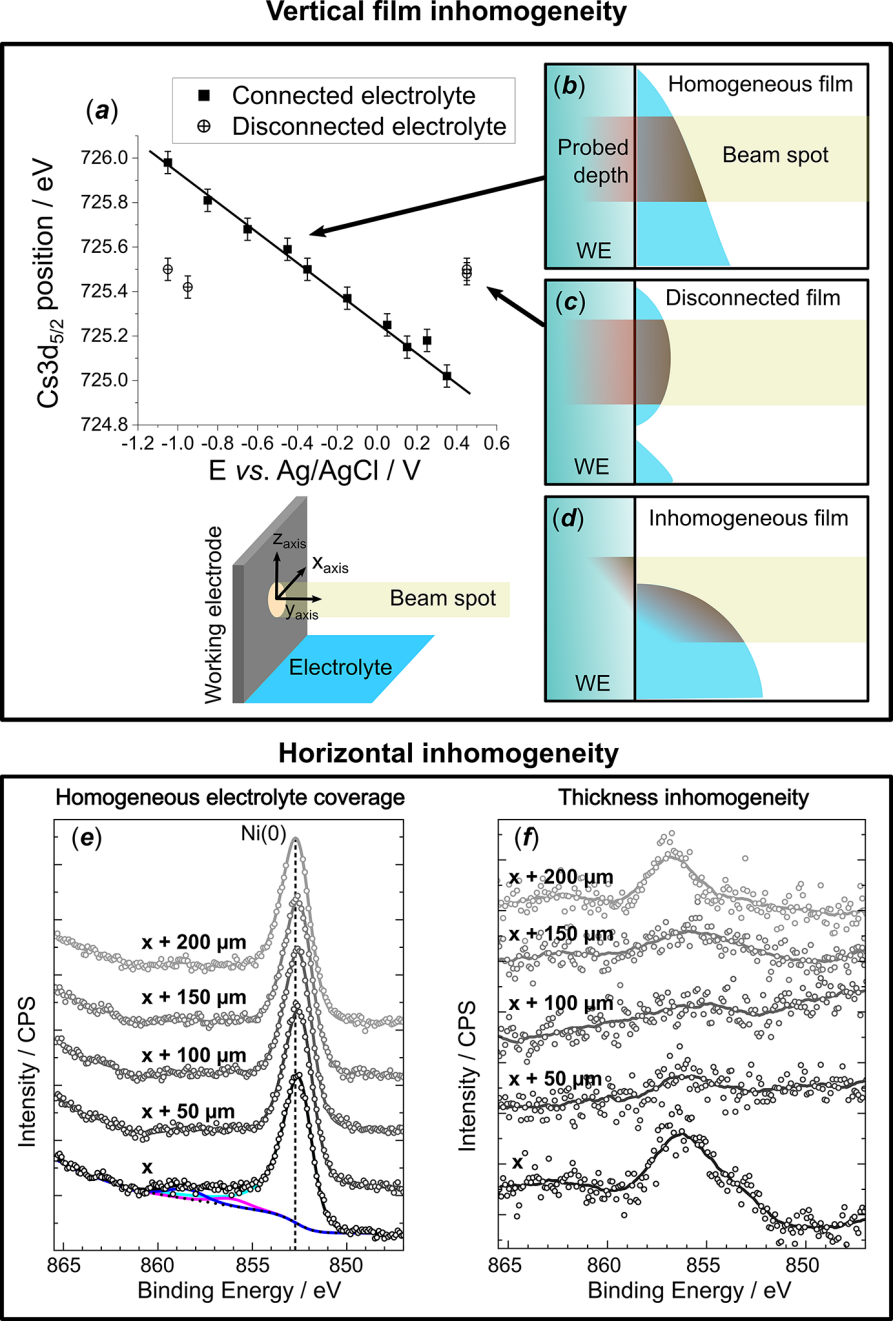
Illustration of the D&P technical challenges related to the inhomogeneities of the thin electrolyte film with (*a*) the XPS (Cs 3*d*) peak shift at the Pt/CsOH 1 *M* interface depending on the applied potential, which can be used as a means of validating an analysis spot electrically connected to the bulk. (*b*–*d*) Possible morphologies of the electrolyte thin film at the analysis spot. (*e*–*f*) Ni 2*p*_3/2_ XPS spectra at a Ni/KOH 0.1 *M* interface on five spots along the horizontal *x* axis separated by 50 µm parallel to the electrode plane: (*e*) was recorded on metallic Ni and illustrates a very reproducible signal over the probed zone; (*f*) was recorded on oxidized Ni and shows the effect of strong inhomogeneities in the film thickness.

As stated above, the thin electrolyte film must not only have an adequate thickness but also be homogeneous. The latter (*i.e.* absence of significant variations in the electrolyte film shape at the analysis spot either vertically or horizontally) is strongly tied to the electrode hydro­philic/hydro­phobic properties. The film homogeneity must be assessed for each electrode composition, which can be done in several steps, as discussed below and illustrated in Fig. 3 ▸ and Fig. S2. First, it is essential to ensure that the beam spot is not positioned at the edge of the electrolyte film [see Fig. 3 ▸(*d*) and Fig. S2(*a*), where part of the electrode is electrolyte-free]. To achieve this, the following protocol is proposed:

(i) Find a spot (spot #A) of a desired electrolyte thickness having a signal from both the electrode and the electrolyte.

(ii) Verify the electrolyte presence right above the analysis spot (*i.e.* by moving the detector vertically by the size of the beam spot, to spot #B).

(iii) Move back to spot #A if electrolyte presence is verified at spot #B and perform a measurement.

If this condition is met, the electrolyte film should be vertically homogeneous [see Fig. 3 ▸(*b*)] in the investigated region. To further probe the homogeneity of the film thickness around the probed spot, several spectra can be acquired along the horizontal *x* axis, parallel to the electrode plane, to ensure that the signal ratio between the electrode and the electrolyte remains constant. If the intensity of the XPS signal of the electrode varies between the spots [as on Fig. 3 ▸(*f*)], it indicates that the film thickness is not constant over the probed zone. This often arises from local electrode surface heterogeneities (*e.g.* the presence of structural defects or oxide islands, the latter either remaining from the preparation procedure or induced by potential gradients during electrochemical oxidation/reduction cycles) which translate into different local hydro­philicities and, thus, different electrolyte film thicknesses and shapes. Ideally, this can be controlled either by moving further in *x* until sequentially acquired spectra become similar [like on Fig. 3 ▸(*e*)] or by dipping the electrode in the electrolyte and pulling it out again.

Finally, the electrolyte presence at the analysis spot does not guarantee electrical connection of the analysed film with the electrolyte bulk, since one cannot discard the formation of electrolyte droplets, disconnected from the bulk [see Fig. 3 ▸(*c*)]. Hence, systematic verification of the shift of the XP peaks arising from electrolyte species with the applied potential, *i.e.* the phenomenon described in the *Introduction* and illustrated in Figs. 1 ▸(*d*) and 1 ▸(*e*), is crucial for confirming that the interface at the probed spot is polarized. This is illustrated on Fig. 2 ▸, with the Cs 3*d* BE_app_ shift as a function of the potential, and rationalized in Fig. 3 ▸(*a*), which gives indications of the electrode polarization at the analysis spot. As mentioned earlier, if the actual potential at the analysed spot is equal to the applied potential, the BE_app_ shift should be equal to −1 eV/V. Yet, deviation from this theoretical slope may be observed for several reasons:

(i) A lower slope [*e.g.* −0.71 eV/V in Fig. 3 ▸(*a*)] can be observed due to ohmic losses in the thin electrolyte film, as a result of the high electrolyte resistance caused by the nanometre thickness of the electrolyte thin film and its centimetre-long shape and the presence of a parasitic Faradaic reaction (Križan *et al.*, 2025 ▸). When considering this in the prism of equation (2), it would affect the electrostatic potential difference (eΔ*V*).

(ii) Faradaic reactions can also influence the BE_app_ shift due to the change in the electron chemical potential between the WE and the photoelectron source Δμ_*e*_ [see equation (2)], as illustrated by Källquist, Ericson *et al.* (2022 ▸) and Källquist *et al.* (2021 ▸). Indeed, in the case of Faradaic reactions, the chemical potential of the electron at the interface can change and this change may mitigate the variation of the electrical potential drop eΔ*V*, as one may see from equation (2).

(iii) If the electrolyte has a low ionic strength, the EDL may exceed the electrolyte thin film thickness and the BE_app_ shift perceived by NAP-XPS may therefore be inferior to the applied potential difference (Lichterman *et al.*, 2017 ▸).

(iv) A point outside the BE_app_/potential trend means a total disconnection from the electrolyte bulk and, thus, a non-polarized analysis spot [see crossed circles on Fig. 3 ▸(*a*)].

(v) RE potential instability during D&P experiments is not unusual due to the low-pressure conditions, resulting in errors in the values of the applied potential. Careful users may avoid such problems by regular *in situ* calibration of the RE during the measurements versus characteristic voltammetric peaks of the WE.

Interestingly, the shift of the XPS peaks induced by the polarization may reach ‘beyond’ the liquid phase, *i.e.* similar shifts are sometimes observed in the BE_app_ of the gas phase with the electrode polarization. Such a shift is much harder to correlate with the applied potential, as the electrical potential is non-uniform in the gas phase. Teschner *et al.* (2024 ▸) provide simulations regarding this shift, highlighting that the electrical potential in the gas strongly depends on the liquid electrolyte film geometry close to the nozzle.

Ultimately, if the interface polarization control is hindered either by (i) ohmic losses, shown by a smaller than 1:1 shift of the BE_app_ of electrolyte species with the applied potential (Križan *et al.*, 2025 ▸), and/or (ii) electrolyte homogeneity issues at the analysis spot, it might be better to switch to a quasi-*in situ* D&P experiment, that is to study a fully emersed electrode. In order to do this (i) the electrode is dipped in the electrolyte reservoir; (ii) the electrochemical conditions of interest are applied to modify the electrode surface chemistry; (iii) the electrode is pulled out of the electrolyte while keeping the polarization (until the loss of electrolytic contact) to position the electrochemically modified part of the electrode in front of the analyser; (iv) XPS measurements are performed on the electrode. This is similar to a *post-mortem* experiment, however, with the advantage of the electrode not suffering from contact with the atmosphere and still being freshly analysed, as the XPS measurement is done in the minute timescale after pulling the electrode out of the electrolyte. Surface chemistry modifications are therefore more likely to persist in the absence of polarization (*i.e.* when the electrode is completely pulled out) until the analysis compared with conventional *post-mortem* assessment. This alternative approach has its roots in the XPS studies of emersed electrodes that were pioneered by Hansen (1983 ▸) and Kötz (1990 ▸), in which samples were studied *ex situ* by transferring the electrode from an electrochemical cell to a vacuum chamber through ambient atmosphere. Later, dedicated transfer systems were introduced, allowing the electrode transfer from an electrochemical cell to the analysis chamber under a controlled atmosphere or vacuum (Wagner & Ross, 1983 ▸). The quasi-*in situ* approach as proposed above has been recently employed by several research groups, for a wide range of applications, such as corrosion and passivity breakdown (Larsson, Simonov *et al.*, 2023 ▸; Larsson, Grespi *et al.*, 2023 ▸), electrocatalysis (Scholten *et al.*, 2019 ▸; Spadetto *et al.*, 2024 ▸) and battery research (Oswald *et al.*, 2009 ▸).

**Figure 4:**
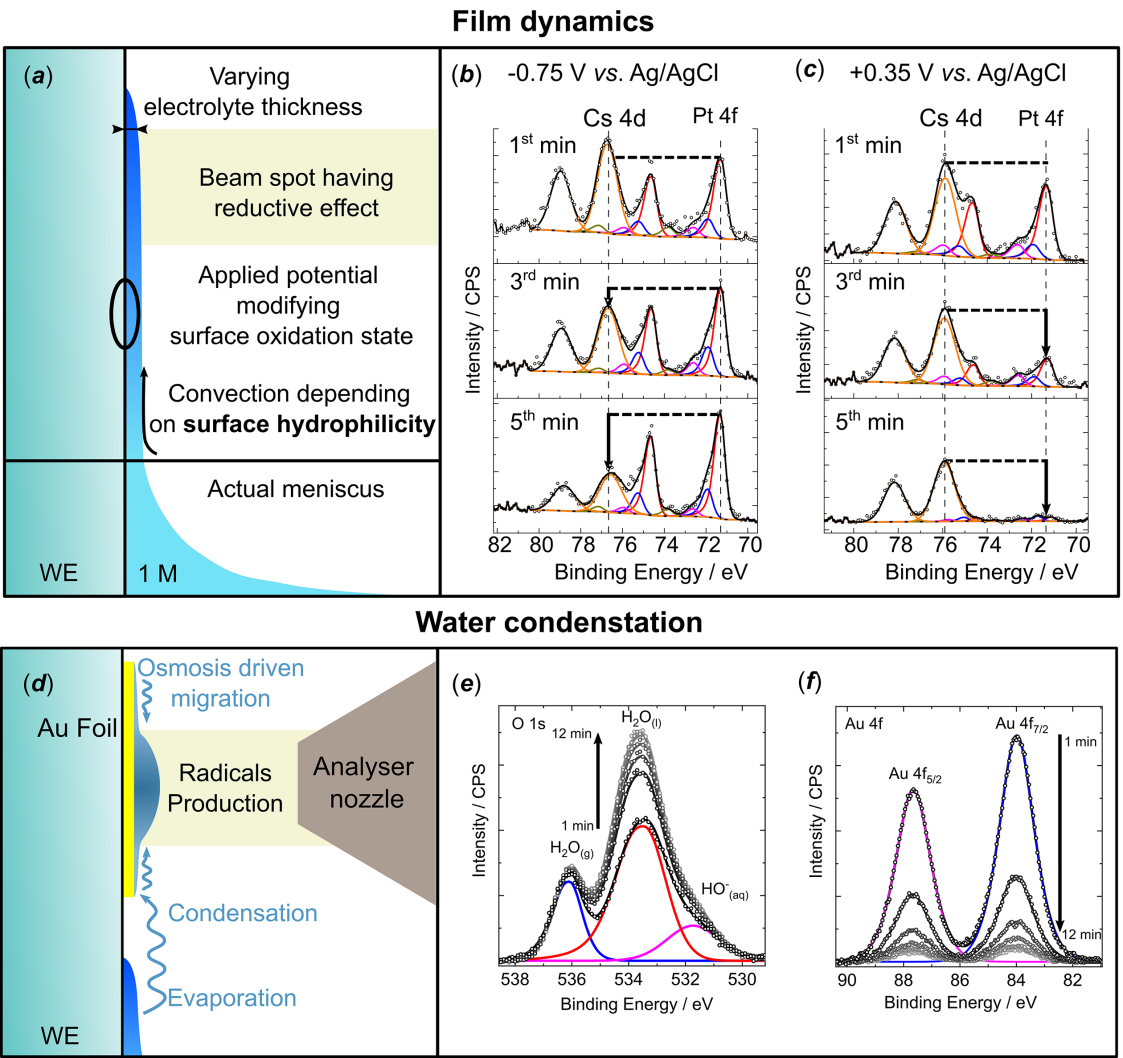
Illustration of the D&P technical challenges associated with the time evolution of the experimental conditions. (*a*) Schematic sectional view of the thin film highlighting the origins of the electrolyte film dynamics during the *in situ*/*operando* experiment; (*b*) and (*c*) are XP spectra of the same Pt/CsOH 1 *M* interface at different times after pulling the electrode from the electrolyte at −0.75 V and +0.35 V versus Ag/AgCl, respectively. *h*ν = 1800 eV, *P* = ∼15 mbar. (*d*) Schematic sectional view highlighting possible accumulation of water in front of the nozzle, presumably due to the local radical production and their dilution by osmosis. (*e*) and (*f*) are successive O 1*s* and Au 4*f* XP spectra of an Au foil, physically disconnected from the electrolyte. *h*ν = 1800 eV, *P* = ∼20 mbar.

Yet another factor to be considered is time. Indeed, due to the (i) beam irradiation, (ii) potential-induced changes in the surface chemistry and (iii) distance between the electrolyte reservoir and the analysis spot, the surface properties and the resulting hydro­philicity evolve as a function of time. The electrolyte thickness can therefore vary during the experiment. This is illustrated on Fig. 4 ▸(*a*) showing XP spectra taken at the Pt/CsOH 1 *M* electrolyte interface, at two different potentials. At −0.75 V versus Ag/AgCl [Fig. 4 ▸(*b*)] the film quickly decreases in thickness, as evident from the increase of the electrode Pt 4*f* contribution compared with the electrolyte Cs 3*d* peaks. In contrast, at higher potential [+0.35 V versus Ag/AgCl, Fig. 4 ▸(*c*)] the Pt 4*f* peak intensity decreases with time until they are ultimately fully suppressed, indicative of an electrolyte film thickness > 3λ_IMFP_. This may be attributed to the increasing surface hydro­philicity upon Pt oxidation evident from an enhancement of the high BE shoulder in Pt 4*f* XP peaks in Fig. 4 ▸(*c*). Beyond variations in the thickness of the film depending on the chemical state of the electrode surface, the former can also change in time due to the solvent condensation, radiolysis or analyser nozzle aspiration. This has been observed on a reference gold foil exposed to a water vapour pressure of ∼20 mbar, as shown on Figs. 4 ▸(*d*)–4 ▸(*f*). Six spectra of Au 4*f* and O 1*s* were recorded successively (∼100 s between two scans), showing a strong decrease of the Au 4*f* intensity, while the intensity of the H_2_O_(l)_ peak increased. Initially, the Au foil was covered by a thin film of liquid water, due to the non-zero pressure inside the analysis chamber. Hence, we speculate that the water accumulation over time may result from beam-induced water radiolysis; this produces radicals at the analysis spot and leads to the accumulation of water locally, owing to the osmotic effect, thus resulting in a quick loss of the Au surface signal due to the water layer thickness increase [see Fig. 4 ▸(*d*)]. Despite the absence of literature reports corroborating the existence of such an effect, the authors believe this ‘radiolysis-driven osmosis’ may be the most likely explanation for accumulation of water under the beam. This effect compensates, and supersedes, the nozzle aspiration effect, which facilitates the electrolyte evaporation locally and induces a local thinning of the electrolyte film over time. To avoid any electrolyte thickness modification between the beginning and the end of the measurement, the most convenient approach is – again – to move along the *x* axis during each scan, although this requires that meniscus horizontal homogeneity is achieved, as illustrated in Fig. 3 ▸(*b*).

**Figure 5:**
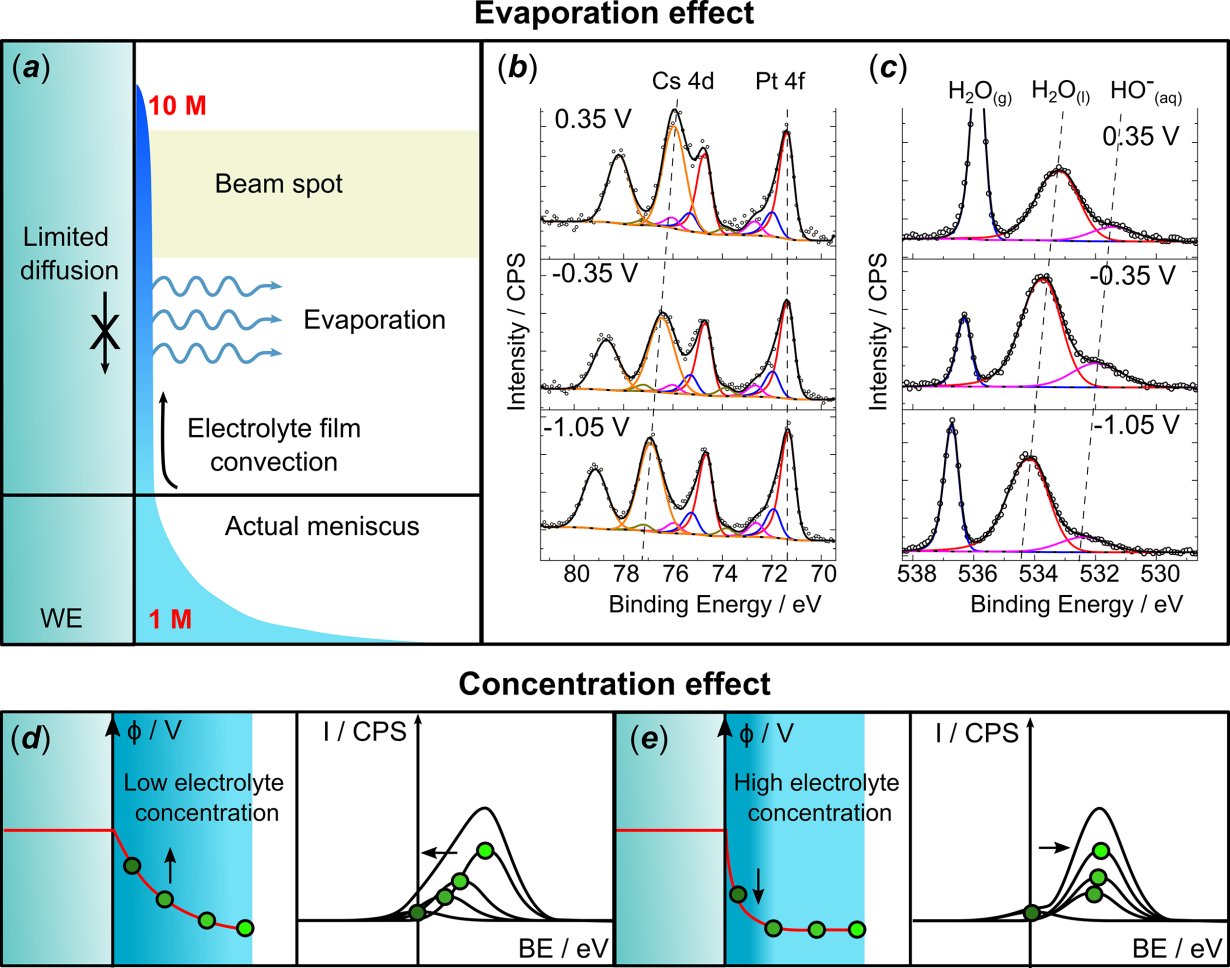
(*a*) D&P electrolyte thin-film schematic sectional view highlighting various reasons for the increase of the electrolyte concentration at the analysis spot; (*b*) and (*c*) are XP spectra of the Pt/1 *M* CsOH electrode–electrolyte interface, respectively, showing the Cs 4*d*, Pt 4*f* and the O 1*s* signal when changing potentials from lower to higher values. The ∼1:5 ratio between H_2_O_(l)_ and HO^−^_(aq)_ corresponds to a ∼10 *M* electrolyte concentration, which was consistent with the Cs 3*d* XPS intensity (not shown here). *h*ν = 1800 eV, *P* = ∼15 mbar. (*d*) and (*e*) are a graphical illustration of the electrolyte concentration in the thin film on the XPS peak shape. Left panels represent the electrical potential drop at the interface, and right panels represent the expected XPS peak shape.

The properties of the electrode/electrolyte interface at the analysis spot are also influenced by the pressure in the analysis chamber. A constant pumping, motivated by the desire to improve the photoelectron intensity, may decrease the pressure below the saturation vapour pressure of the electrolyte [*e.g.* ∼31 mbar for pure water at 25°C (Lide, 2004 ▸)] and lead to its evaporation over time. Given the geometry of the electrolyte thin film, water evaporation from the electrolyte is favoured, leading to a substantial concentration increase at the analysis spot. Indeed, the HO^−^/H_2_O_(l)_ ratio estimated from the respective components of the O 1*s* XP spectra [see Fig. 5 ▸(*c*)] was similar to 1:5, which corresponds to ∼10 *M* actual CsOH concentration instead of 1 *M* used for the experiment. This high concentration is consistent with the equilibrium vapour pressure for a CsOH solution in the conditions reached during this experiment (15 mbar) (Balej, 1985 ▸). Such an increase of the electrolyte concentration can substantially affect the conditions at the electrode/electrolyte interface, by modifying the surface chemistry and decreasing the EDL thickness, therefore limiting our capability to assess potential and ion distribution in the latter, as illustrated in Figs. 5 ▸(*d*)–5 ▸(*e*). To limit the evaporation and avoid the concentration increase within the meniscus, controlling pressure in the analysis chamber at the level corresponding to the saturated water vapour pressure is essential, in order to work as much as possible at the thermodynamic equilibrium conditions between liquid and vapour. However, other conditions being equal, increasing the pressure in the analysis chamber significantly decreases the intensity of the measured XPS signal and therefore increases the time required to record a spectrum, which limits the applicability of D&P to *operando* systems. Decreasing the electrolyte reservoir temperature using a cooling system was proposed by Larsson, Simonov *et al.* (2023 ▸) to limit the evaporation in the electrolyte reservoir. However, this adaptation only affected the bulk of the electrolyte, and had little impact on the thermodynamic equilibrium at the thin film/gas interface and, thus, on the evaporation from the electrolyte thin film.

Finally, diffusion through the two-dimensional thin electrolyte film can be an issue (Favaro, 2020 ▸). The local consumption of dissolved species at the interface modifies the local concentration of reactants and products more drastically than in the electrolyte bulk. In particular, the study of the electrode/electrolyte interface during electrochemical reactions involving water molecules, protons and/or hydroxyl ions (*e.g.* oxygen evolution reaction – OER, hydrogen evolution reaction – HER) adds a further challenge due to the changes of local pH, resulting from hindered electrolyte replenishment if protons or hydroxyls are consumed/produced (Favaro, 2020 ▸). Furthermore, as highlighted above, the soft and tender X-ray induced photoemission process is often accompanied by radiolysis, triggering ionic fragmentation of molecules and/or interfacial oxidation degree changes (Buxton, 2020 ▸; Ferradini & Jay-Gerin, 1999 ▸; Weatherup *et al.*, 2018 ▸). For instance, water radiolysis produces solvated electrons, H^**.**^ and OH^**.**^ radicals, as well as H_3_O^+^, H_2_ and OH^−^ which can react with surrounding species, modify the pH or the oxidation degree of elements at the interface (Schneider *et al.*, 2014 ▸; Cheng *et al.*, 2024 ▸). Depending on the experimental conditions, such radiolysis effects can be quite significant in soft and tender X-ray studies (Weatherup *et al.*, 2018 ▸; Arble *et al.*, 2020 ▸; Schneider *et al.*, 2014 ▸). Since the radiolysis effect is related to the local concentration of radiolysis products that are formed at the analysis spot, mitigating the beam radiolysis effect can therefore be done by (i) moving the incident X-ray beam out of the focus, (ii) diminishing its overall intensity at the cost of longer acquisition time, (iii) designing spectro-electrochemical cells with an electrolyte flow to replace the electrolyte damaged by irradiation (which is however not feasible for the D&P configuration), or (iv) moving horizontally along the *x* axis between each scan to probe a non-irradiated area [see Fig. 3 ▸(*e*)] (Arble *et al.*, 2020 ▸). However, this lateral motion should consider the diffusion of the radiolysis species during the spectra acquisition, implying that the shift performed between every 10 min-long acquisition should be larger than 250 µm (this for a diffusion coefficient of ∼10^−6^ cm^2^ s^−1^).

In conclusion, a ‘good’ D&P experiment must involve the following steps: (i) identification of a meaningful analysis spot (*i.e.* avoiding the thin electrolyte film edge vertically and ensuring a homogeneous film of an adequate thickness and connected to the electrolyte bulk at distances along the *x* axis significantly exceeding dimensions of the analysis spot), and (ii) monitoring its stability, which often requires displacement of the analysis spot in between the scans along the vertical *z* axis (due to the film dynamics), and horizontal *x* axis (owing to the nozzle aspiration effect and beam-induced modification of the electrolyte). This, however, does not suffice to make the conditions at the analysis spot ideal from an electrochemical standpoint. They differ from the bulk conditions, owing both to the evaporation effect (*i.e.* substantial changes in the electrolyte concentration at the analysis spot) and to ohmic losses in the meniscus. As demonstrated in Fig. 3 ▸(*d*), the BE_app_ versus the applied potential slope is usually below 1 eV/V, indicating that the potential ‘seen’ at the analysis spot differs from the applied potential. Furthermore, as shown by stochastic simulations of Favaro *et al.*, diffusion in a thin liquid electrolyte layer primarily occurs along the direction orthogonal to the interface, but is greatly impeded in the direction parallel to the WE surface (Favaro, 2020 ▸), thus limiting applicability of the method to Faradaic reactions accompanied by consumption of reactant/generation of products (where diffusion plays an essential role), and making it better adapted to studies of non-Faradaic reactions (charge/discharge) or adsorption/desorption.

This being said, with experimental rigour, one can gather meaningful insights into the electrode (Han *et al.*, 2018 ▸; Favaro, Valero-Vidal *et al.*, 2017 ▸; Favaro, Drisdell *et al.*, 2017 ▸; Favaro, Yang *et al.*, 2017 ▸; Ali-Löytty *et al.*, 2016 ▸; Larsson, Simonov *et al.*, 2023 ▸) or the electrolyte properties (Capone *et al.*, 2024 ▸; Källquist *et al.*, 2021 ▸; Wibowo *et al.*, 2023 ▸; Temperton *et al.*, 2022 ▸) with the D&P method. Applicability of the method to studies of the EDL will be analysed in the next section.

## 4. On the applicability of the D&P approach to EDL studies

**Figure 6:**
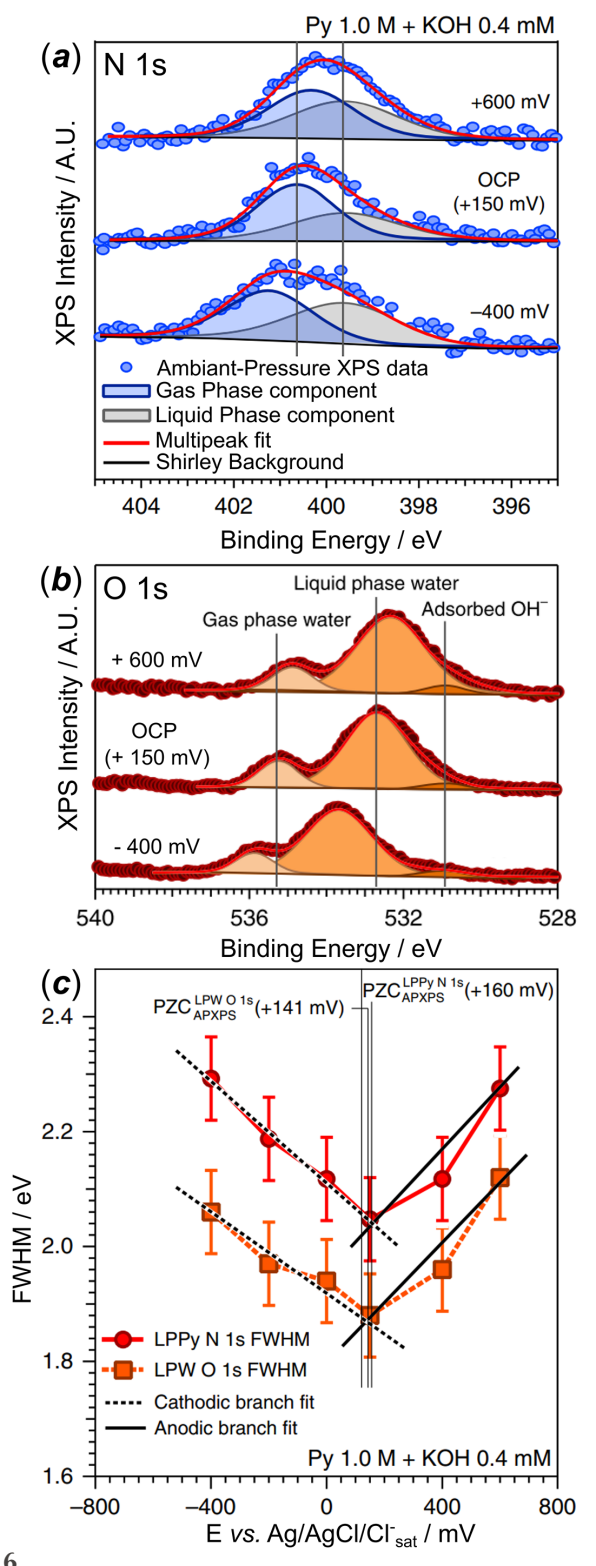
EDL study in KOH 0.4 m*M* + 1 *M* pyrazine by D&P. Adapted from Favaro *et al.* (2016 ▸). (*a*, *b*) N 1*s* and O 1*s* XPS signal depending on the applied potential, (*c*) FWHM of O 1*s* liquid phase water component depending on the applied potential.

Favaro *et al.* (2016 ▸) were the first to publish a proof of concept that highlighted the possibility of assessing the EDL by the D&P method. To do so, they followed the solvent (H_2_O_(l)_) and a molecular probe (pyrazine) XPS signal (N 1*s* and O 1*s*) for a gold WE in 0.4 m*M* KOH + 1 *M* pyrazine electrolyte, depending on the applied potential [Figs. 6 ▸(*a*), 6 ▸(*b*)], and plotted the full width at half-maximum (FWHM) of those peaks at different applied potentials [Fig. 6 ▸(*c*)]. At the PZC, the ionic double layer does not build up at the interface, and the FWHMs of the O 1*s* and N 1*s* peaks reach a minimum, but increase when the potential moves away from the PZC. This observation was made possible by applying (i) a low (0.4 m*M*) KOH concentration in the electrolyte, (ii) a high chamber pressure (*i.e.* 30 mbar, leading to little to no increase of the salt concentration in the electrolyte thin film), and (iii) a highly concentrated probe (pyrazine). Another attempt to ‘observe’ the EDL by D&P was made by Lichterman *et al.* (2017 ▸) who used Ir (WE) in a KOH electrolyte of concentrations ranging from 3.2 × 10^−3^ *M* to 1 *M*, and varied the electrolyte film thicknesses at the analysis spot from 18 to 42 nm. The objective was to quantitatively estimate the influence of these experimental variables on the XP spectra, particularly on key metrics such as the electrolyte peak shifts and their FWHM. The authors experimentally demonstrated the influence of pH and electrolyte thickness on the BE_app_ shift of the O 1*s* XPS peak. Indeed, in the case of ‘low’ electrolyte concentration (3.2 × 10^−3^ *M* KOH) the EDL thickness was found to be greater than the actual electrolyte thickness in the thin film at the electrolyte spot, resulting in a low BE_app_ shift (∼−0.6 eV/V), since only a part of the bulk electrode/electrolyte potential drop could be probed. In contrast, the EDL was far thinner than the electrolyte thickness in 1 *M* KOH, resulting in the expected −1 eV/V shift for the same film thickness. Specifically, the authors estimated a ∼2.5% change in the FWHM value with a film thickness of 10 nm at 1 *M* electrolyte concentration, while a 32% change may be expected at 3.2 × 10^−3^ *M* KOH with a similar film thickness. Those values do not consider the likely increase of the concentration in the meniscus owing to low operating pressures (∼15–20 mbar), that will further (i) increase the overall XPS signal intensity but (ii) decrease the FWHM variations.

One may thus identify two different approaches to investigate the EDL: (i) through monitoring non-charged molecular probes and solvent molecules [similar to the work by Favaro *et al.* (2016 ▸) and Lichterman *et al.* (2017 ▸)], or (ii) through probing ions that are involved in the EDL formation. As the characteristic EDL thickness decreases with the ionic strength of the solution, working with non-charged probes remains the most straightforward solution, as the probe concentration can be increased while not influencing the EDL thickness and still allowing one to gather insights into key properties such as the electrode PZC. However, measuring the signal of charged species would provide information on their distribution depending on the distance to the electrode, *i.e.* on the structure of the EDL itself. This is however more challenging due to the EDL thinning at high ion concentration. When the nature of the ionic or molecular probe is not imposed by experimental necessity, one could benefit from choosing elements with a high cross section value. The latter can be found in several sources, the most recent one being the calculations from Trzhaskovskaya & Yarzhemsky (2018 ▸, 2019 ▸), Willis *et al.* (2020 ▸). Overall, the cross section increases with the *Z* element number, while also depending on the type of transition. In alkaline media, higher signal intensity is expected using Cs^+^ rather than Li^+^, Na^+^ or K^+^ counterparts [the photoionization cross section σ at 2 keV photon energy increases from Li^+^ to Cs^+^ as follows: σ(Li 1*s*) = ∼0.26 × 10^−21^ cm^2^; σ(Na 2*s*) = ∼2.6 × 10^−21^ cm^2^; σ(K 2*s*) = ∼16 × 10^−21^ cm^2^, σ(Cs 3*d*_5/2_) = ∼130 × 10^−21^ cm^2^]. Alternatively, the incident photon energy can also be tuned. It directly affects the probing depth of the XPS: low energies are suitable for surface studies, while high energies are suitable for in-depth probing. For example, in several publications (Favaro *et al.*, 2016 ▸; Stoerzinger *et al.*, 2018 ▸; Lichterman *et al.*, 2017 ▸; Favaro, Yang *et al.*, 2017 ▸) a ∼4 keV beam energy was used to analyse a sample below a ∼35 nm-thick electrolyte film. Under such a configuration, the electron photoemission from a Pt 4*f* peak [considering λ_IMPF_ in water at 3.9 keV of ∼12 nm, estimated with *Quases-IMFP-TPP2M* 3.0 (Tougaard, 2021 ▸) and coherent with the work of Shinotsuka *et al.* (2017 ▸)] is reduced to 5.4% of the electrolyte-free intensity, while using 1.8 keV beam energy (λ_IMFP_ in water for a 1.7 keV photoelectron is ∼6 nm) for a similar electrolyte thickness reduces the signal to 0.3% of the electrolyte-free intensity. This highlights how the contribution of the species close to the interface and thus the EDL can be emphasized by increasing the photon energy. Another solution when studying species at the liquid–solid interface in a D&P system is to enhance the experimental approach with computational tools. Thus, recently, Qian *et al.* (2020 ▸) proposed an approach to connect electronic structure and chemical information from *ab initio* molecular dynamics, density functional theory and analytical model calculations to the experimentally obtained XP spectra with the objective of facilitating the assessment of experimentally observed features, especially when considering signals of interfacial species ‘buried’ under a liquid layer of ∼10 nm.

## 5. Conclusions and perspectives

Since its introduction in 2015, the D&P method has shown promise for studying electrochemical systems under *in situ* and *operando* conditions. As it uses model WEs with quasi-2D planar geometry, and since photoelectrons travel through a thin electrolyte layer covering the WE, this approach offers new opportunities and enhanced sensitivity to the electrolyte part of the interface; this has not been possible in the ‘transparent-window’ configuration cells. These various opportunities are not, however, free of challenges. Indeed, one of the strengths of XPS, the surface sensitivity, is also one of its main constraints, as it limits the thickness of the electrolyte layer in the D&P configuration at the analysis spot to <35 nm for photon energies around 4 keV. Furthermore, establishing an ‘ideal’ electrolyte thin film and maintaining it throughout the measurement is challenging. The electrolyte film may exhibit heterogeneities (*e.g.* vertical, horizontal, disconnection from the electrolyte bulk) at the start of the experiment, but may also evolve during measurements (*e.g.* due to a change in surface hydro­philicity, radiolysis, analyser nozzle aspiration or, simply, evaporation). Some of these phenomena can be alleviated by applying rigorous experimental protocols (*e.g*. identifying changes and moving the analysis spot) or switching from *in situ*/*operando*, to quasi-*in situ* conditions (*i.e.* dip the electrode, apply the potential and pull out the electrode after a given time, while breaking its contact to the electrolyte), to ensure that the analysis spot is exposed to the desired conditions prior to the analysis. A combination of these two approaches allowed us, as well as other research groups, to gather significant insights into the evolution of the electrolyte and/or electrode chemical composition and oxidation state under an applied potential.

This article provides elements to understand the particular behaviour of the electrolyte film within the analysis spot in the D&P setup. Although centralized around alkaline electrolytes, most of the phenomena and methodologies discussed here remain valid when switching to aqueous acidic or organic media:

(*a*) In an acidic environment, behaviour associated with water radiolysis and evaporation is expected to be similar, while the EDL would be even harder to follow due to the lack of a good XPS marker to track, owing to the lower cross section of C, Cl, P, B, S *etc.* compared with cations with high atomic numbers.

(*b*) Using organic electrolytes with a higher boiling point in the D&P setup alleviates some of the technical limits due to their lower evaporation rate. However, (i) water radiolysis is replaced by organic radiolysis – whose effect on the electrochemical system may be even harder to comprehend – and (ii) the higher viscosity of the solvent would modify the behaviour of the meniscus. Following the methodology proposed herein to verify the electrolyte thin-film homogeneity and to avoid complex radiolytic degradation effects could therefore be even more important.

In a nutshell, the exact physico-chemical behaviour of the system depends on the unique experimental conditions applied – from the electrochemical system to the spectrometer. It is therefore essential for the user to adopt a rigorous approach and an open mind with respect to the variety of phenomena that may occur in the D&P setup discussed here or yet to be observed.

## Supplementary Material

Supporting Information, providing a detailed description of the dip-and-pull procedure and validation means used within this work. DOI: 10.1107/S1600577525008811/ye5070sup1.pdf
