# Supplementary material for: Methodological insights into the dip-and-pull X-ray photoelectron spectroscopy technique: analysing electrochemical interfaces under in situ/operando conditions
Source: J Synchrotron Radiat. 2026 Jan 1;33(Pt 1):130–41. doi: 10.1107/S1600577525008811 (PMC12809443; doi:10.1107/S1600577525008811)
Supplement: Supplementary file 1 [file s-33-00130-sup1.pdf]

JOURNAL OF SYNCHROTRON RADIATION

Volume 33 (2026)

Supporting information for article:

Methodological insights into the dip-and-pull X-ray photoelectron spectroscopy technique: analysing electrochemical interfaces under *in situ/operando* conditions

Benjamin Rotonnelli, Amandine Brige, Alexandr G. Oshchepkov, Jean-Jacques Gallet, Fabrice Bournel, Antoine Bonnefont, Alexander Yaroslavtsev, Andrey Shavorskiy, Robert Temperton, Elena R. Savinova and Tristan Asset

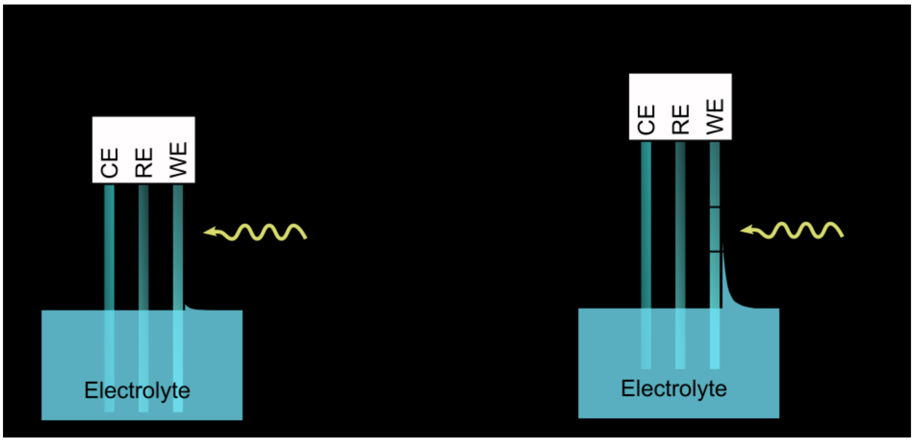

Figure S1: Illustration of the experimental protocol. **A)** Before the acquisition of a new experimental point, the electrode is dipped into the electrolyte bulk, **B)** then polarised, and **C)** finally pulled out of the electrolyte until the electrolyte XPS signal is detected at the analysis spot with the desired ratio of intensity between the electrode and electrolyte XPS signals.

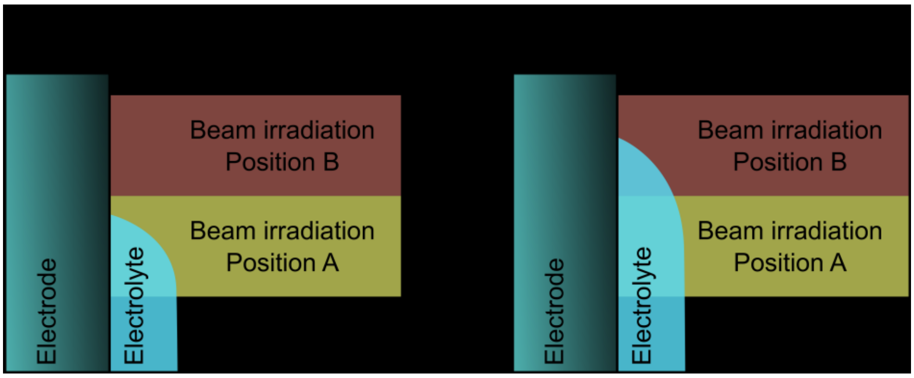

Figure S2: Once an area displaying the signal of the electrode and of the electrolyte is found, the user must verify the good homogeneity of the electrolyte thin-film, through the verification of electrolyte presence within the beam spot B, just above the desired analysis spot (spot A). **Case A)** if the electrolyte thin film ends abruptly in the middle of spot A, spot B should not exhibit the electrolyte presence. **Case B)** If the electrolyte is still observed on the spot B, this should mean that electrolyte thin-film is present within the full height of the spot A.

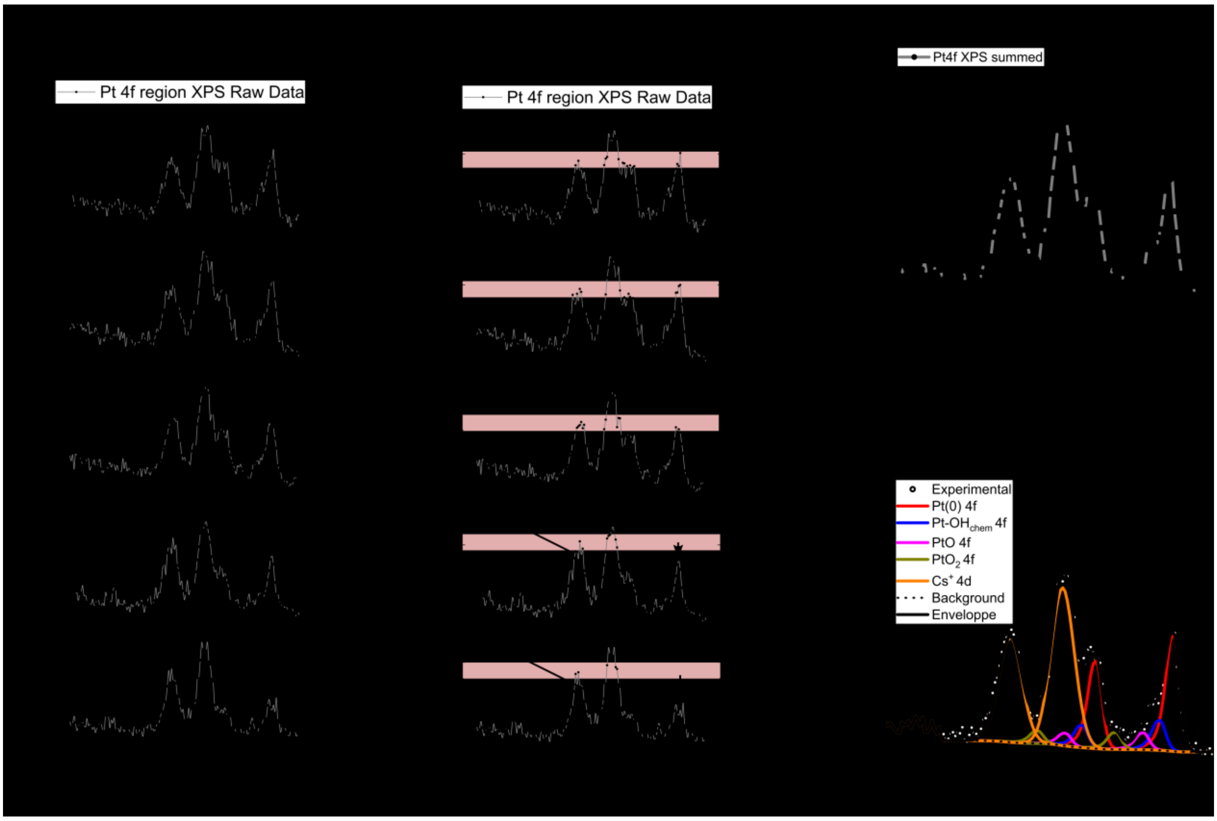

Figure S3: Illustration of the method adopted for the data acquisition and validation in the dip-and-pull setup. **A)** As soon as a valid analysis spot is found (**Figures S1** and **S2**), several short XPS scans are acquired (in this case: 5 scans with ~30 s acquisition per scan and per region). **B)** In order to obtain a meaningful spectrum that is representative of the applied polarisation, the XPS signal must remain stable. Each scan must therefore be compared each other to ensure that they are similar and avoid changing the experimental environment during the data acquisition process (*e.g.* electrolyte thickness evolution, polarisation loss, *etc.*). In the shown example, a latitude of +/- 10% was tolerated in the normalised intensity evolution between Cs 4ds 3/2 and Pt 4f 5/2, which is illustrated with the red band. *The definition of the tolerance criterion must be adapted for each experiment.* **C)** The scans that show a good consistency are summed up, and **D)** data treatment can begin with the data obtained from these signals.

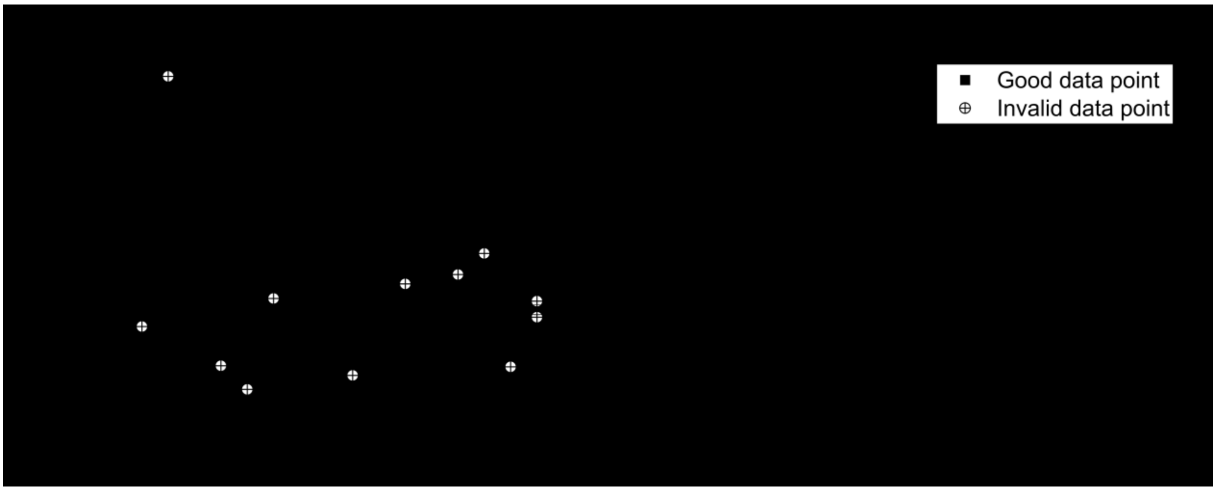

Figure S4: Once the full data set has been checked and fitted, further verifications are required to ensure the consistency of conditions in the full experimental data set. **A)** The consistency of the electrolyte thin film thickness in the dataset can be judged from the ratio of the electrode XPS signal area (here, the Pt 4f component), and the electrolyte XPS signal area (here, the $H_2O_{(l)}$ O 1s component). In our case, a threshold was set to remove data that did not match the desired electrolyte thickness. In addition to the electrolyte thickness verification, **B)** the polarisation of the interface is evidenced by the regular shift of the XPS peak from electrolyte species depending on the applied potential. Only those experimental spectra **that passed both** of the aforementioned checks were analysed and discussed.
